# The FDA MyStudies app: a reusable platform for distributed clinical trials and real-world evidence studies

**DOI:** 10.1093/jamiaopen/ooaa061

**Published:** 2020-12-11

**Authors:** Zachary Wyner, Sascha Dublin, Christina Chambers, Shyam Deval, Chayim Herzig-Marx, Shanthala Rao, Adam Rauch, Juliane Reynolds, Jeffrey S Brown, David Martin

**Affiliations:** 1 Dana-Farber Cancer Institute, Boston, Massachusetts, USA; 2 Kaiser Permanente Washington Health Research Institute, Kaiser Permanente Washington, Seattle, Washington, USA; 3 Department of Pediatrics, University of California, San Diego, La Jolla, California, USA; 4 Boston Technology Corporation, Boston, Massachusetts, USA; 5 Division of Therapeutics Research and Infectious Disease Epidemiology, Department of Population Medicine, Harvard Pilgrim Health Care Institute, Boston, Massachusetts, USA; 6 LabKey, Seattle, Washington, USA; 7 Moderna, Inc, Cambridge, Massachusetts, USA

**Keywords:** mobile app, digital health, patient-generated data, mobile devices, open source, web-based configuration, real-world evidence, clinical trials, research studies, ResearchKit, ResearchStack

## Abstract

We developed a mobile application and secure patient data storage platform, FDA MyStudies, to address privacy, engagement, and extensibility challenges in mobile clinical research. The system extends the capabilities of the mobile frameworks Apple ResearchKit and ResearchStack through an intuitive front-end application and secure storage environment that can support health research studies. The platform supports single or multisite studies via role-based access and can be implemented within highly secure data environments. As a proof-of-concept, pregnant women participated in a descriptive study via the app in which data not routinely captured in electronic health records (EHR) were collected and linked with existing patient data to provide a more wholistic view of the patient and illustrate how patient data combined with EHR data could be used to support public health research.

## BACKGROUND AND SIGNIFICANCE

Despite the promise of “Big Data,” most comparative effectiveness and drug safety research in the United States relies on electronic health records (EHR) and health care claims data. However, these data sources do not reliably capture information critical to the conduct of such studies such as medication adherence, exercise, health outcomes that occur outside the medical home, and use of vitamin, supplement, and over-the-counter medications. In addition, these electronic data sources often do not reliably capture the patient perspective, which is critical for endpoints such as pain, quality of life, disease progression, and functional status. To fill these gaps, clinical trials or prospective studies that include patient-reported information are often needed. Mobile apps have the potential to increase efficiency in such prospective studies.

Existing mobile frameworks like Apple ResearchKit and ResearchStack provide a foundation on which to efficiently build and distribute mobile-based studies. A PubMed search on the keywords ResearchKit and ResearchStack identified 10 studies conducted using an app built on the Apple ResearchKit framework.^1–11^

Still, challenges remain using existing mobile frameworks, especially for studies intended for regulatory purposes, in areas such as (1) consent, privacy, and security; (2) compliant enrollment and sustained engagement; and (3) extensibility and support of multiple therapeutic areas within a single mobile app framework.^12^^,^^13^ To address these challenges, the Food and Drug Administration (FDA) engaged Harvard Pilgrim Health Care Institute (HPHCI) through the FDA Sentinel Initiative Catalyst program to coordinate the development and pilot testing of the FDA MyStudies platform.^14^^,^^15^

The primary goal of the project was to build an open-source reusable platform comprised of a mobile device application and patient data storage environment that addresses the challenges noted above in a manner consistent with FDA’s regulatory needs regarding data security and traceability. Creating a platform that meets regulatory data security and privacy standards while remaining extensible to different types of studies and patient cohorts was central to the requirements and design of the platform.

As a proof-of-concept, we conducted a descriptive pilot study of exposures and health outcomes among pregnant women from the Kaiser Permanente Washington (KPWA) health system. Full pilot study objectives, methods, and results are reported elsewhere in a brief communication pending publication. The FDA MyStudies platform is generalizable and can be used and rebranded for studies in other therapeutic areas and patient populations and has recently been modified to enable contactless patient informed consent during the COVID pandemic.^16^

In this article, we describe the capabilities of the platform, how it expands the usability of existing mobile frameworks, and how it can help form a wholistic view of study participants. Open source code and technical documentation are available to the public on GitHub.

## MATERIALS AND METHODS

### Requirements gathering

A workgroup consisting of members from FDA, Boston Technology Corporation (BTC), LabKey, Kaiser Permanente Washington Health Research Institute (KPWHRI), Vaccines and Medications in Pregnancy Surveillance System (VAMPSS), and HPHCI developed the mobile app platform business requirements. The workgroup also incorporated feedback from other FDA stakeholders to ensure the technical and hosting architecture was consistent with emerging FDA standards for mobile applications. During development, the KPWHRI team led focus groups that included seven women who were pregnant or had been pregnant within the past three years to provide feedback on a prototype system.

### Design

**Figure 1:**
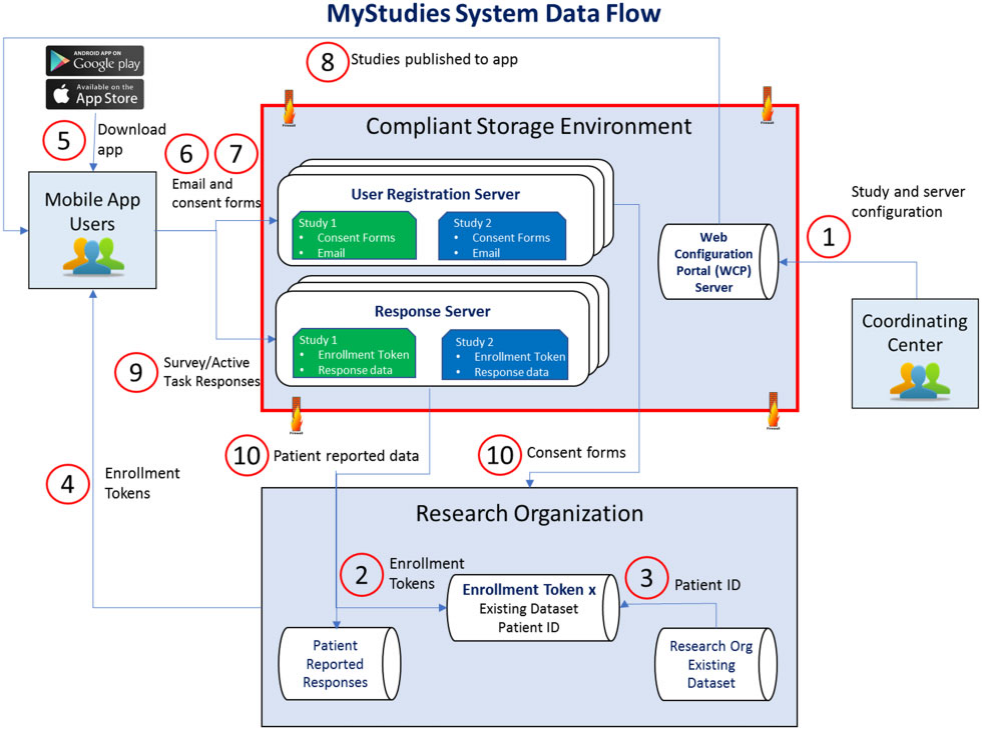
Business context diagram.

The FDA MyStudies platform contains three elements: (1) an iOS and Android version of the mobile app, (2) a web-based configuration portal (WCP) for configuring studies and other elements accessed via the apps, and (3) a secure storage environment to manage study participant responses and consent forms. Applications developed under the FDA Sentinel Initiative must adhere to these security regulations, but the platform code can be implemented in environments with less stringent security requirements. Multiple apps, rather than a responsive design, were required to enable custom enhancements to the ResearchStack frameworks. Figure 1 illustrates how all system elements interact.

The storage environment consists of three independent servers: a response server to store data captured via the mobile app, a registration server to store participant information (email addresses and consent forms), and a metadata server for information configured in the WCP. This design was chosen to enable partitioning of patient identifiable information from response data. A unique set of credentials is required to access each server; there is no method to match email addresses and consent forms to response data without access to both relevant servers.

The response and registration servers are custom implementations of the LabKey Server product.^17^ Two add-ons were built for this project: (1) a module that automatically creates a new database schema for every new questionnaire, eliminating the need to manually create a schema for each new study; (2) the ability to produce a unique token, or enrollment token, which can be given to participants to restrict enrollment to a specified cohort or match data to external data sets. All three servers enforce role-based governance.

The platform stores data in a centralized location and does not interact directly with an EHR system as that represented a policy change outside the scope of this project.

Study materials, including questionnaire content, consent forms, eligibility questions, and app notifications, are configurable and distributed to the apps in real time via the WCP. All Apple ResearchKit and ResearchStack question types can be used in a questionnaire. Participants respond to questionnaires at their convenience and responses are stored locally until submission or questionnaire expiration.

The mobile app and WCP were released to production in September 2017 and made publicly available via GitHub in November 2018.

## RESULTS

### Enrollment

The app was published in the Apple App Store and Google Play Store immediately before pilot study invitations were mailed to 1071 pregnant women who met minimum inclusion criteria. 81 Apple iOS users and 38 Android users downloaded the FDA MyStudies app and a total of 64 (6%) patients consented via the mobile app to participate in the study.

**Figure 2:**
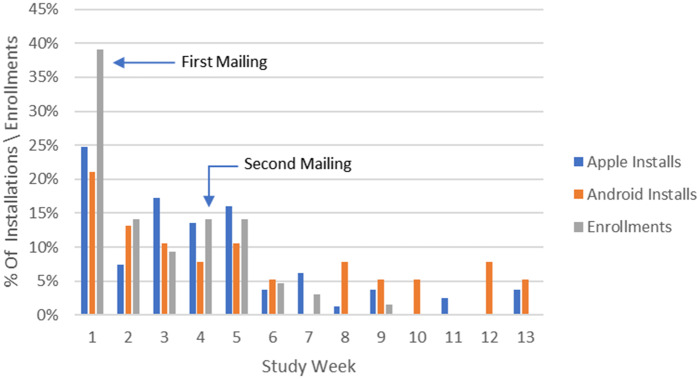
Installations and enrollments over time.

Figure 2 shows the percent of those users that installed the app each week. 39% enrolled in week 1, 14% in week 2, 9% in week 3, and 14% in week 4. The slight increase in enrollment in week 4 is likely due to the second set of invitation letters sent out at that time.

### Engagement

The pilot study collected data for 13 weeks. Half of the cohort (51%) engaged with the app for more than 4 weeks and the median period of engagement was 35 days. Table 1 shows the completion percentage for each survey distributed during the pilot study.

**Table 1:** Questionnaire completion by name

Questionnaire name	Women who completed at least 1 distribution of the questionnaire	%	Number of times completed^a^
	*N* = 64^a^		
Current weight^b^	59	92	264
Initial study questionnaire	59	92	59
Medical condition history	58	91	58
Pregnancy status^b^	58	91	288
Short-term illness history during pregnancy	53	83	54
Pregnancy history	50	78	50
Information about you	48	75	48
Smoking and vaping history	45	70	45
Vaccine history during pregnancy	44	69	44
Vitamin use history	40	63	40
Current medical condition information	39	61	106
History of alcohol exposure	39	61	39
History of marijuana or cannabis exposure	39	61	39
History of recreational drug exposure	39	61	39
Recent short-term illnesses^b^	36	56	92
Current smoking and vaping exposure^b^	31	48	45
Current vitamin use^b^	30	47	44
Current alcohol exposure^b^	26	41	26
Current marijuana or cannabis exposure^b^	26	41	26
Current recreational drug exposure^b^	26	41	26
Vaccine exposure update^b^	13	20	14
Current pregnancy outcomes^b^	7	11	7

a Three women did not answer any questionnaires.

b Recurring questionnaire.

## DISCUSSION

### Extensibility and usability

The MyStudies platform is an extensible publicly available mobile data collection tool designed to support research studies intended for regulatory use. Other mobile apps designed for research are typically purpose built for one study and require programming expertise to reconfigure for new use cases; the MyStudies platform is designed to enable rapid redeployment for different studies leveraging the same underlying platform.

By adding a front-end application (*ie* the WCP) and secure storage environment to existing mobile frameworks, the platform allows simple user interface-based configuration, mobile patient consent, and secure data storage in a single platform.

In the pilot study, KPWHRI was able to link each woman’s unique enrollment token with her patient record in the KPWA EHR using a crosswalk file stored externally to the EHR. This linkage allowed patient-reported data from the app to be compared with data recorded in the EHR and to augment the EHR data to better support research studies; due to health system data governance requirements data from the app were not directly incorporated into the EHR but rather linked to EHR-derived research data set stored outside the EHR. Patient-reported information such as over-the-counter medication use, use of alcohol, and illicit drugs can fill common gaps in the EHR, thereby better enabling EHR-based research to support study aims. The platform now allows the pushing of data directly to external systems such as EHRs.

### Consent, privacy, and security

Mobile app uptake for research is a function of the effectiveness of the initial recruitment method used, possession of a smartphone capable of operating the app, and a willingness to download the app, authenticate, provide electronic consent, and use the app. The system conforms to stringent federal data privacy and security standards. The LabKey storage environment ensures data backup services, encryption, and log monitoring of the are enabled.^17^ The secure enrollment token process ensures indefinable information is not stored in the same server as patients’ responses.

Third-party review of the system for 21 CFR Part 11 compliance was beyond the scope of the project, but we are confident that the data storage environment is secure and supports auditing necessary for compliance with 21 CFR Part 11;^18^ the system was designed and built with that goal in mind via input from FDA stakeholders. Two-factor authentication (via email) is used during registration and app users can be authenticated by investigators if needed by matching email address to responses. Electronic signatures are required during each study consent process and the enrollment token ensures only the selected cohort can join. Signed consent forms are saved as PDFs in the storage environment and accessible to investigators with appropriate permissions.

### Limitations

Use of the app requires individuals to have an Android or iOS-compatible mobile device, which may influence the composition of the study population. However, smartphone and tablet usage is widespread and growing^19^ and it is possible to combine the app-based study approach with alternative approaches such as a web-based survey instrument. We note that some patient populations, for example, those who are unable to engage with a mobile app due to their disease state or who are uncomfortable with the technology, may not be good candidates for this data collection and engagement approach.

The study had a fixed start date and survey schedule, so some questionnaires expired before all patients were enrolled. This potentially influenced engagement as not all participants had the opportunity to complete all questionnaires. The newest version of the app now available on GitHub allows for staggered enrollment which addresses this limitation.

Lastly, the system is designed to be downloaded from GitHub and used independently of the existing FDA infrastructure built for the pilot. Therefore, some engagement with software developers may be required to locally install the system and rebrand the app code with specific branding desired by an organization and study. An Apple and/or Google app developer account is also required to distribute the app. The compliance version of the LabKey server is required to ensure all security features described but a free version of the LabKey server is available. Implementation of new studies using the app should plan several months of preparation for each new study, depending on the study design and complexity of the questions. Still, all elements of the system are designed to work seamlessly together to provide an intuitive and compliant mobile data collection platform that supports multiple therapeutic areas in a single app. The HPHCI workgroup members and the technical partners are using the MyStudies app to support multiple clinical studies in different disease areas and have recently released a version to support Covid-19 clinical trials.^17^^,^^20^ In addition, Google is now working on a 1-click implementation for the app.^21^

## CONCLUSION

The pilot study demonstrated that the FDA MyStudies platform can be used to collect data directly from patients while meeting the current challenges presented by mobile apps with regards to (1) consent, privacy, and security; (2) enrollment and engagement; and (3) extensibility and represents a substantial expansion to the usability of existing mobile frameworks.

Given the scope of information provided, the app appears capable of expanding the depth and diversity of patient-reported information available to support clinical research, either alone or in combination with other electronic data sources such as EHRs, claims data, and registries. Continued app development using the information in the public domain will likely increase usability, enable the integration of other real-world data sources, and enhance the overall ease with which the patient perspective can be included in clinical research. This work fills helps to address a critical gap by enabling investigators and policy makers to incorporate the patient voice in critical public health research.

## Authors’ contributions

Each author made substantial contributions to the study design, study implementation, and analysis, and each contributed to drafting, reviewing, and approving the final manuscript.

## Notes

This article reflects the views of the authors and should not be construed to represent FDA’s views or policies.
